# Nutcracker Syndrome Complicated by Left Renal Vein Thrombosis

**DOI:** 10.1155/2013/168057

**Published:** 2013-11-24

**Authors:** Faouzi Mallat, Wissem Hmida, Mehdi Jaidane, Nadia Mama, Faouzi Mosbah

**Affiliations:** ^1^Department of Urology, Hospital of Sahloul, 4054 Sousse, Tunisia; ^2^Department of Radiology, Hospital of Sahloul, 4054 Sousse, Tunisia

## Abstract

Isolated renal vein thrombosis is a rare entity. We present a patient whose complaint of flank pain led to the diagnosis of a renal vein thrombosis. In this case, abdominal computed tomography angiography was helpful in diagnosing the nutcracker syndrome complicated by the renal vein thrombosis. Anticoagulation was started and three weeks later, CTA showed complete disappearance of the renal vein thrombosis. To treat the Nutcracker syndrome, we proposed left renal vein transposition that the patient consented to.

## 1. Introduction

Renal vein thrombosis is a fairly uncommon site for vascular occlusion when isolated to this location. Cases of solitary renal vein thrombosis have been reported to be in association with the nutcracker syndrome [1, 2]. Here, we present an intriguing case in which a 24-year-old female with no previous major health problems; especially she has no known history of coagulopathy, presented with acute flank pain.

During the course of her evaluation, a computed tomography (CT) of the abdomen and pelvis revealed unilateral renal vein thrombosis with anterior nutcracker syndrome. 

## 2. Case Report

We present the case of a 24-year-old newly married woman, who was admitted to our hospital from the Emergency Department complaining of acute and constant left flank pain for 3 days with macroscopic haematuria. 

This pain was nonradiating, was not related to meals, did not respond to over-the-counter analgesics, and was not associated with anorexia, nausea, vomiting, or fever. This pain was preceded by macroscopic haematuria that appeared for 2 months before.

Personal and family history was noncontributory; especially she denied any history of surgeries, chronic illness, recent immobilisation, recent prolonged travel, or trauma or oral contraceptives. She was not pregnant. No thrombogenic factors were identified.

She reported no change in her bowel habits.

The patient's vitals were stable and exam revealed isolated left costovertebral angle tenderness. Physical examination revealed also a height of 172 cm and weight of 51 kg with a lower BMI at 17.2. 

Her initial labs revealed only haemoglobin at 9.0 mg/dL. 

**Figure 1:**
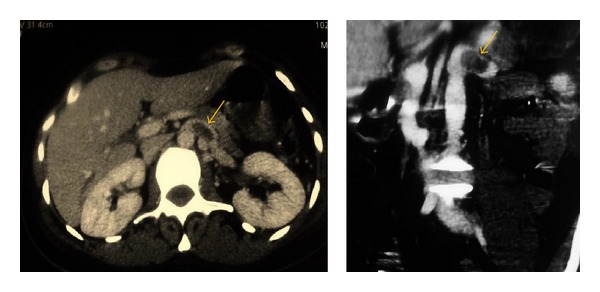
CT of the abdomen and pelvis demonstrating a dilated left renal vein with intraluminal thrombus (yellow arrow).

**Figure 2:**
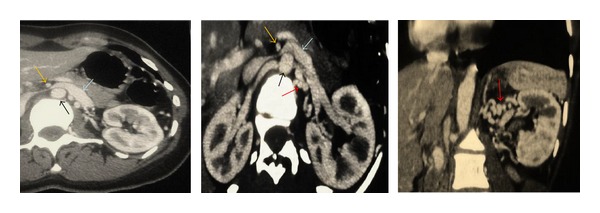
CTA of the abdomen and pelvis showed complete disappearance of the renal vein thrombosis and demonstrated the nutcracker syndrome: compression of the LRV between the aorta (black arrow) and the SMA (yellow arrow), with a dilatation of the hilar portion of the LRV (blue arrow). The red arrow showed and surrounding vascular collaterals.

Computed tomographic angiography (CTA) was performed demonstrating left renal vein thrombosis (Figure 1), regardless of renal tumor or hydronephrosis. The CTA also showed a compression of the left renal vein (LRV) between the aorta and the superior mesenteric artery (SMA), SMA angle (the angle between aorta and SMA) was approximately 10°, the posteroanterior diameter of the hilar portion and that of the aorticomesenteric stenotic portion of the LRV were 9.9 mm and 1.0 mm, respectively (Figure 2), and the diagnosis of anterior nutcracker syndrome leading to left renal vein thrombosis was confirmed.

When we asked her again, she reported a long history of various symptoms including intermittent macroscopic haematuria and chronic moderate left lumbar pain aggravated by physical activity, associated with systemic signs dominated by chronic fatigue and headache, which lasted for his childhood, despite a long investigational history combined with several imaging examinations and laboratory tests. Urinalysis revealed 4+ proteinuria, and 24 hour urine collection analysis showed 2 g of proteinuria.

Based on clinical presentation, urinary and systemic symptoms, laboratory reports, and computed tomographic angiography finding, a diagnosis of anterior nutcracker syndrome leading to left renal vein thrombosis was confirmed.

Anticoagulation was started by a low molecular weight heparin bridge to warfarin. Three weeks later, CTA showed complete disappearance of the renal vein thrombosis (Figure 2).

The patient was informed of her diagnosis and there was a discussion about possible treatment options. We proposed transposition of left renal vein that the patient consented to. 

 Since she was newly married, it was decided to initially bridge her to warfarin with plans for 6 months of anticoagulation followed by repeated imaging. Need for additional management would be decided at that time.

## 3. Discussion

To the best of our knowledge, we present the third case of thromboembolic complication of this syndrome in the literature and describe the left renal vein thrombosis as a circumstance of discovery of the nutcracker syndrome.

 Renal vein thrombosis is a rare entity that is usually associated with a chromogenic background. Renal vein thrombosis associated with NCS is a rare entity, and only 2 cases were found in a review of the literature [1, 2]. 

Known as Virchow's triad, the three broad categories of factors thought to contribute to thrombosis are mural factors, coagulation factors, and blood stasis. 

Our present patient had no prothrombotic coagulation factors and no determinable mural factors or related inflammation as seen in thrombophlebitis, such as Buerger's disease. Blood congestion caused by the Nutcracker syndrome may therefore have contributed to the pathogenesis in our patient.

 Anterior nutcracker syndrome results from compression of the left renal vein between the superior mesenteric artery and the aorta, with subsequent development of venous varicosities of the renal pelvis, ureter, and the gonadal vein [3]. The true prevalence of nutcracker syndrome remains unknown and it might be underdiagnosed.

The NCS is characterized by impeded outflow from the left renal vein (LRV) into the inferior vena cava due to extrinsic LRV compression, accompanied by LRV hypertension [3]. 

Because the diagnosis of NCS is commonly delayed, as seen in our patient despite a long investigational history combined with several imaging examinations and laboratory tests, the chronic blood congestion in the left renal vein may therefore have contributed to the pathogenesis of the thrombosis. 

The higher sensitivity and specificity of CTA make this the procedure of choice in the diagnosis of the renal vein thrombosis and may have a role in differentiating this condition in woman presenting with acute left lumbar pain. CTA helps to reveal the thrombus extension and help to find aetiology such as the nutcracker syndrome especially in young females. 

Management of the renal vein thrombosis due to NCS aims to treat the thrombus, prevent pulmonary embolism, and treat the cause of the thrombus. 

With regard to the renal vein thrombosis management, a satisfactory outcome with anticoagulation therapy alone was obtained [1, 2], as seen in our case. 

 Treatment of the NCS is controversial. Different treatment options have been proposed for this syndrome, including followup, conservative treatment, and surgical therapy. 

The available surgical procedures aim to decrease LRV hypertension; they include intra- or extravascular stents and open surgical procedures employed to rectify the problem including the transposition of left renal vein, transposition of the superior mesenteric artery, renal autotransplantation, and nephropexy [3, 4]. The indications for laparoscopic surgery continue to augment for the treatment of the nutcracker syndrome: retroperitoneal laparoscopic nephrectomy with ex vitro autograft repair and autotransplantation [5], laparoscopic splenorenal venous bypass [6], laparoscopic extravascular renal vein stent placement [7], and laparoscopic inferior mesenteric-gonadal vein bypasses [8].

 The interventions should be considered only when symptoms are severe or persistent, including severe unrelenting pain, severe hematuria, renal insufficiency, and failure to respond to conservative treatment [3]. Because the symptoms of NCS in our patient are severe and complicated by renal vein thrombosis, we proposed transposition of left renal vein that the patient consented to. 

## 4. Conclusion

Patients with NCS are at an increased risk of venous thrombosis. This is essentially due to the presence of persistent blood congestion. In a patient with NCS, the appearance of sudden unexplained flank pain should raise suspicion of renal vein thrombosis.
